# Serum Vascular Endothelial Growth Factor Levels before and after Intravitreous Ranibizumab Injection for Retinopathy of Prematurity

**DOI:** 10.1155/2019/2985161

**Published:** 2019-05-20

**Authors:** Xuting Chen, Lin Zhou, Qi Zhang, Yu Xu, Peiquan Zhao, Hongping Xia

**Affiliations:** ^1^Xinhua Hospital, Department of Neonatology, Shanghai Jiao Tong University School of Medicine, Shanghai, China; ^2^Xinhua Hospital, Department of Ophthalmology, Shanghai Jiao Tong University School of Medicine, Shanghai, China

## Abstract

**Background:**

Retinopathy of prematurity (ROP) is one of the common complications of prematurity. Intravitreal injection of ranibizumab (IVR), an antivascular endothelial growth factor (VEGF) drug, showed significant benefit for ROP. However, there are concerns about systemic complications of anti-VEGF therapy in preterm infants.

**Objectives:**

To evaluate serum VEGF level in the systemic circulation after IVR and the complications associated with IVR for the premature infants with ROP.

**Methods:**

This prospective investigation assessed the serum concentrations of VEGF in ROP patients before and after IVR therapy. All the infants had binocular retinopathy and received IVR 0.25 mg per eye as the primary treatment. Serum samples were collected 1 day prior to injection and 1 day, 3 days, and 7 days after IVR treatment. Serum VEGF level was measured by the enzyme-linked immunosorbent assay (ELISA).

**Results:**

Fifteen infants (6 girls and 9 boys) were enrolled. The serum concentrations of VEGF 1 day before and 1 day, 3 days, and 7 days after a total of 0.5 mg intravitreal injections of ranibizumab were 226.9 (198.4, 272.4), 12.8 (7.0, 22.4), 16 (12.0, 20.8), and 33.7 (24.0, 48.0) pg/ml, respectively. Serum VEGF levels decreased significantly at 1 day, 3 days, and 7 days after IVR treatment compared with pretreatment concentration (*P* < 0.05). Compared to days 1 and 3 after IVR, serum VEGF level at 7 days after IVR treatment increased significantly (*P* < 0.05).

**Conclusion:**

Serum VEGF levels in patients with ROP were suppressed for at least 7 days after IVR treatment. Although the clinical significance of this phenomenon is uncertain, its safety profile requires further investigation.

## 1. Introduction

Retinopathy of prematurity (ROP) is a leading cause of blindness in children around the world. Suppression of growth factors secondary to hyperoxia and loss of the maternal-fetal interaction result in an arrest of retinal vascularisation. Subsequently, the increasingly metabolically active, yet poorly vascularised, retina becomes hypoxic, stimulating growth factor-induced vasoproliferation, which can ultimately result in retinal detachment and blindness [1, 2]. Vascular endothelial growth factor (VEGF), a central mediator of angiogenesis, is considered to play an important role in the pathogenesis of ROP [3, 4]. Recent systemic review suggests that intravitreal anti-VEGF agents reduce the risk of refractive errors (high myopia) during childhood compared with laser therapy in infants with type 1 ROP [5]. The largest anti-VEGF study for ROP to date, the BEAT-ROP (efficacy of intravitreal bevacizumab for stage 3 + retinopathy of prematurity) trial, found that bevacizumab, a full-size anti-VEGF antibody, can halt the progression of severe ROP, revert pathologic angiogenic changes, and induce the progression of physiologic intraretinal vasculature [6].

However, VEGF is important in normal angiogenesis in the early newborn period, and the aim of anti-VEGF treatment is to selectively block the excessive levels of VEGF trapped within the overlying vitreous and not to penetrate tissue. Preterm infant with proliferative ROP has a compromised blood-retinal barrier possibly allowing a large amount of VEGF inhibitors to enter blood stream, so systemic side effects are of particular interest. A single dose of intravitreally injected bevacizumab suppresses VEGF plasma levels below the limit of detection for weeks [7]. In adult patients with age-related macular degeneration, with respect to systemic VEGF suppression, ranibizumab may be a safer alternative to bevacizumab [8, 9]. There is little evidence on the safety of intravitreal injection of ranibizumab (IVR) in preterm infants with ROP. The result of the RAINBOW study, an ongoing international multicenter trial about efficacy and safety of IVR, is still unknown. The aim of this study is to evaluate the serum VEGF level in the systemic circulation after IVR and the complications associated with IVR for the premature infants with ROP.

## 2. Materials and Methods

### 2.1. Study Population

This prospective investigation assessed the serum concentrations of VEGF in ROP patients before and after IVR therapy. The fundus of preterm infants was examined after pupillary dilation using binocular indirect ophthalmoscopy under topical anesthesia, and fundus photographs were obtained with a RetCam digital fundus camera. The stage of the ROP was based on the International Classification of Retinopathy of Prematurity [10]. All of the diagnoses were confirmed independently by at least two ophthalmologists who had sufficient knowledge and experience in treating ROP. All the infants who had binocular retinopathy with type 1 ROP received IVR as the primary treatment to prevent macular dragging and tractional retinal detachment. Type 1 ROP was defined as stage 1 or 2 ROP in zone I with plus disease or stage 3 ROP in zone I, or stage 2 or 3 ROP with plus disease in zone II, based on the Early Treatment for Retinopathy of Prematurity Randomized Trial study [11]. And none of the infants underwent laser photocoagulation of the peripheral avascular retina before IVR. This study was conducted from June 2017 to July 2018. The status of the off-label use of IVR for ROP treatment was explained to the parents of the patients in detail. All the parents of the patients provided informed consent before the administration of IVR, and written informed consent was obtained from the parents for enrollment of their kids in the study. Adverse events reported with the systemic administration of anti-VEGF monoclonal antibodies, including thromboembolic events, myocardial infarction, stroke, kidney disease, hypertension, and gastrointestinal perforations, were monitored for after IVR treatment in these patients. This study was approved by the Ethics Committee of Xinhua Hospital, Shanghai Jiao Tong University School of Medicine and was registered in ClinicalTrial.gov (ID: NCTO3115255).

### 2.2. Intravitreal Injection of Antivascular Endothelial Growth Factor Drugs

All the infants had binocular retinopathy. The eyes were prepared in a standard fashion using 5% povidone/iodine and topical antibiotics. For each eye, 0.25 mg ranibizumab (0.025 ml) (equivalent to 50% of the standard adult dose) (Lucentis; Genentech Inc, South San Francisco, CA, US) was injected intravitreally 1.5 mm posterior to the limbus via the pars plicata under intravenous sedation. The injection was performed initially with a 30-gauge needle directed perpendicularly to the globe and then directed slightly toward the center of the globe after the tip of the needle passed the lens equator. Care was taken to prevent damaging the lens or retina. After the injection, retinal artery perfusion was checked, and the patients received the topical antibiotic levofloxacin three times a day for three days.

### 2.3. Vascular Endothelial Growth Factor Measurement

Blood samples were collected at 1 day prior to IVR and at 1, 3, and 7 days after IVR therapy. After collection, the blood samples were centrifuged at 3,000 rpm for 10 minutes. The serum was transferred to sterile tubes and stored at −20°C until analysis.

### 2.4. ELISA

The serum VEGF concentration in study patients with ROP was quantified using a commercially available ELISA kit for human anti-VEGF (Human VEGF Immunoassay; R&D Systems, Minneapolis, MN), which was able to detect the 121 and 165 isoforms of VEGF according to the manufacturer's protocol. VEGF concentration was calculated according to the standards used as protein-adjusted amount of VEGF. The minimum detectable level of the test was 9.0 pg/ml.

### 2.5. Recurrences and Follow-Ups

Recurrence was defined as the return of vascular dilation and tortuosity and the stage 3 disorder in zone 1 or 2. Patients were followed-up at 1 day, 3 days, and 7 days and then weekly or biweekly or monthly after IVR treatment. The endpoint of follow-up was complete involution of acute-phase ROP (neovascularisation and plus disease) with vascularisation of zone III, but not necessarily with vessels reaching temporal ora serrata [10, 12]. Extensive follow-up was individually tailored according to their response to treatment.

### 2.6. Statistical Analysis

Continuous variables were presented as mean ± SD or median (interquartile range (IQR)). Differences in medians were assessed using the Mann–Whitney *U* test between two groups and the Kruskal–Wallis test among three or four groups to compare differences of serum VEGF levels at each time point. Statistical analysis was performed using SPSS software (version 20.0; SPSS Inc, Chicago, IL, USA). Significance was defined as *P* < 0.05.

## 3. Results

Fifteen infants (6 girls and 9 boys) with ROP who received IVR were enrolled in the study. The demographic characteristics and serum VEGF concentrations of the infants are summarized in Tables 1 and 2. The mean gestational age of the infants was 29.10 ± 1.05 weeks, and the mean birth weight was 1243.60 ± 198.45 grams. The mean postmenstrual age at initial IVR was 35.87 ± 2.14 weeks. No obvious adverse systemic complications attributable to IVR were noted in 7 days after IVR in these infants, including myocardial infarction, stroke, kidney disease, hypertension, and gastrointestinal perforations. No new intraventricular hemorrhage, sepsis, and respiratory failure were observed in 7 days after IVR either. Retinopathy prematurity recurrence requiring retreatment occurred in 33.3% of the enrolled patients (5 of 15 with bilateral). Diode laser ablation was given to the avascular retina. All recurrences were resolved at the last visit without adverse anatomic outcomes like macular ectopia, dragged disc, or retinal detachment.

**Figure 1:**
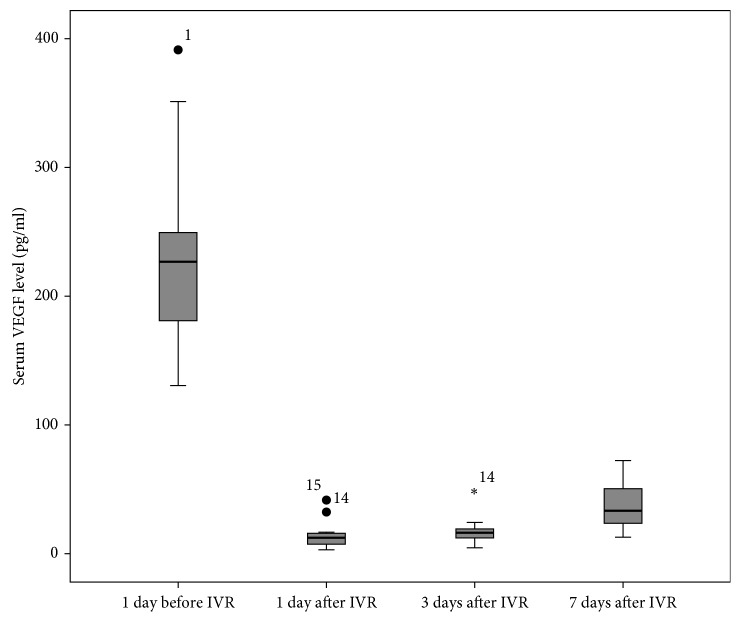
Time course of serum VEGF level in infants with ROP who received a total of 0.5 mg of intravitreal ranibizumab. The abscissa represents the time point. ^*∗*^*P* < 0.05.

The serum VEGF concentrations at 1 day prior to IVR and 1 day, 3 days, and 7 days after IVR were 226.9 (198.4, 272.4) pg/ml, 12.8 (7.0, 22.4) pg/ml, 16 (12.0, 20.8) pg/ml, and 33.7 (24.0, 48.0) pg/ml, respectively. Serum VEGF concentrations decreased significantly at 1 day, 3 days, and 7 days after IVR compared with that concentration 1 day prior to IVR therapy (*P* < 0.05). Serum VEGF level at 7 days after IVR increased significantly compared with that at 1 day or 3 days after IVR (*P* < 0.05). There was no significant difference in the VEGF level between 1 day and 3 days after IVR (*P* < 0.05) (Figure 1).

## 4. Discussion

In this study, we found a significant reduction in serum VEGF levels after a one-time dose of 0.5 mg of intravitreal ranibizumab in preterm infants with ROP. At 7 days after IVR, serum VEGF level was still significantly lower than that prior to IVR therapy in these infants.

VEGF is vital in angiogenesis, in maintaining organ health, in repairing wounds after injury, and in the development of various vital organs in the body. The inhibition of VEGF raises concerns that these important physiologic effects associated with VEGF will be inhibited, possibly leading to abnormal organogenesis or neurodevelopment [13]. After intravitreal injection, VEGF inhibitors may enter the systemic circulation and decrease systemic VEGF levels. Sato et al. found that bevacizumab could escape from the eye into the systemic circulation and reduce the serum VEGF level at least 2 weeks after intravitreal injection of bevacizumab (IVB) in infants with ROP [7]. Wu et al. also showed that bevacizumab was in the systemic circulation 1 day after IVB, and VEGF was suppressed for 2 months [14]. Hong et al. found that IVB reduced plasma VEGF in infants with threshold ROP over a 7-week period [15]. Ranibizumab is a Fab molecule with small size and has short systemic half-life, which is different from bevacizumab [16–18]. In contrast to bevacizumab, ranibizumab has no Fc portion which is typically recycled by binding to neonatal Fc receptor (FcRn) of endothelial cell to prevent it from entering the degradative pathway within endosomes and thereby increasing systemic clearance of ranibizumab [19]. In 2013, Hoerster et al. reported that serum VEGF levels decrease post bilateral injection of 0.2 mg of ranibizumab reaching a nadir around 2 weeks and return to normal levels 4 weeks after injection in an extremely premature infant with a gestational age of 22.7 weeks [20]. By contrast, Zhou et al. found that IVR reduced plasma VEGF levels 1 day after injection in eleven infants with ROP, and this phenomenon disappeared 1 week after the injection [21]. Wu et al. reported that the serum VEGF level in four patients with type 1 ROP was 357.7 (162.7–704.1) pg/ml at 2 weeks after IVR treatment compared with 351.8 (281.9–634.4) pg/ml before IVR [22]. In our study, the results showed that serum VEGF levels in patients with ROP were suppressed for at least 7 days after IVR treatment. The different results in different studies may be related to different doses of ranibizumab, different postmenstrual age of the infants, and the different blood sample selection.

For the anti-VEGF treatment of ROP, half the adult dose per eye is most frequently used. However, the lowest dose with effective effect and less systemic toxicity is unknown. Wallace et al. conducted a masked, multicenter, and phase 1 study to determine whether a much lower dose of bevacizumab than previously used might be effective in treating severe ROP with the aim of reducing systemic risk without compromising benefit, and the result showed that a dose of bevacizumab as low as 0.031 mg was effective in 9 of 9 eyes with type 1 ROP [23]. Recently, CARE-ROP study also showed that the plasma VEGF levels were not systematically altered after 0.12 mg or 0.2 mg ranibizumab injections per eye in the infants with bilateral ROP, and the lower-dose ranibizumab appeared to be as effective as the other [24]. Both doses are lower than 0.25 mg per eye which is most frequently used in ROP therapy.

There were several limitations in this study. The sample size was relatively limited. The blood specimens were collected within 7 days after IVR because of difficulty in obtaining blood samples from the premature infants. We could not determine the exact time that the serum VEGF level returned to baseline concentration from this study. Although no short-term systemic adverse events were observed in our patients, long-term evaluation of infants is warranted for possible effects after intravitreal ranibizumab in ROP patients. Based on the current data, the selection of an anti-VEGF agent with less-systemic VEGF interference or reducing the dose of ranibizumab injection in ROP patients seems to be a prudent choice.

## 5. Conclusion

Serum VEGF levels in patients with ROP were suppressed for at least 7 days after IVR treatment. Although the clinical significance of this phenomenon is uncertain, its safety profile requires further investigation.

## Figures and Tables

**Table 1:** Demographic characteristics of the infants with ROP.

Patient	Sex	Gestational age (week)	Birth weight (g)	Postmenstrual age at IVR (week)	Eye	Zone/stage	Plus disease	ROP regression	Recurrence
1	Female	30	1380	35^+3^	Right	II/1	+	Yes	No
					Left	II/1	+	Yes	No
2	Male	30^+2^	1400	39^+6^	Right	II/2	+	Yes	No
					Left	II/2	+	Yes	No
3	Male	29^+4^	1100	37	Right	II/2	+	Yes	No
					Left	II/2	+	Yes	No
4	Female	29^+6^	1300	36^+3^	Right	AP-ROP		Yes	No
					Left	AP-ROP		Yes	No
5	Female	28^+4^	950	35^+6^	Right	II/1	+	Yes	No
					Left	II/1	+	Yes	No
6	Female	27^+5^	1280	32^+5^	Right	AP-ROP		Yes	Yes
					Left	AP-ROP		Yes	Yes
7	Male	28^+2^	950	36^+5^	Right	II/2	+	Yes	Yes
					Left	II/2	+	Yes	Yes
8	Male	28^+4^	1029	34^+1^	Right	AP-ROP		Yes	Yes
					Left	AP-ROP		Yes	Yes
9	Male	29^+3^	1500	34	Right	AP-ROP		Yes	No
					Left	AP-ROP		Yes	No
10	Male	27^+6^	1200	32^+2^	Right	AP-ROP		Yes	No
					Left	AP-ROP		Yes	No
11	Male	30^+1^	1300	38^+3^	Right	AP-ROP		Yes	Yes
					Left	AP-ROP		Yes	Yes
12	Female	28^+5^	1310	38^+3^	Right	II/2	+	Yes	No
					Left	II/3	+	Yes	No
13	Male	27^+1^	980	36^+6^	Right	II/2	+	Yes	Yes
					Left	II/2	+	Yes	Yes
14	Female	30	1465	35^+3^	Right	I/2	+	Yes	No
					Left	I/2	+	Yes	No
15	Male	30^+3^	1510	34^+3^	Right	I/2	+	Yes	No
					Left	I/1	+	Yes	No

**Table 2:** Serum VEGF levels in infants with ROP.

Patient	Serum VEGF levels (pg/ml)			
	1 day before IVR	1 day after IVR	3 days after IVR	7 days after IVR
1	391.57	12.75	15.97	22.41
2	149.77	7.91	9.52	17.58
3	238.80	12.75	15.97	72.09
4	185.86	22.41	9.52	NA
5	215.51	9.52	4.68	54.51
6	295.84	NA	25.63	28.85
7	350.75	15.97	12.75	41.69
8	337.08	NA	19.19	33.67
9	249.04	3.07	22.41	25.63
10	226.88	15.97	11.14	24.02
11	138.73	6.3	15.82	33.67
12	248.09	6.3	24.02	60.91
13	224.84	52.91	19.05	NA
14	210.84	31.94	48.11	48.04
15	130.84	41.69	15.97	12.59
Median (IQR)	226.9 (198.4, 272.4)	12.8 (7.9, 22.4)	16.0 (12.0, 20.8)	33.7 (24.0, 48.0)

NA: the serum VEGF level is not available because of the limited sample volumes.

## Data Availability

The data used to support the findings of this study are included within the article.
